# Evaluating the effectiveness of GP endorsement on increasing participation in the NHS Bowel Cancer Screening Programme in England: study protocol for a randomized controlled trial

**DOI:** 10.1186/1745-6215-13-18

**Published:** 2012-02-20

**Authors:** Sarah Damery, Steve Smith, Alison Clements, Roger Holder, Linda Nichols, Heather Draper, Sue Clifford, Laura Parker, Richard Hobbs, Sue Wilson

**Affiliations:** 1 Primary Care Clinical Sciences, University of Birmingham, Edgbaston, West Midlands, B15 2TT, UK; 2 Midlands and North West Bowel Cancer Screening Hub, University Hospitals Coventry and Warwickshire NHS Trust, University Hospital, Clifford Bridge Road, Coventry, CV2 2DX, UK; 3 Department of Primary Health Care Sciences, University of Oxford, 2nd Floor, 23-38 Hythe Bridge Street, Oxford, OX1 2ET, UK

**Keywords:** Colorectal cancer, General practitioner, Screening, FOBt, Uptake, Non-responder, Randomised controlled trial, Qualitative, Endorsement

## Abstract

**Background:**

The success and cost-effectiveness of bowel cancer screening depends on achieving and maintaining high screening uptake rates. The involvement of GPs in screening has been found to improve patient compliance. Therefore, the endorsement of screening by GPs may increase uptake rates amongst non-responders.

**Methods/Design:**

A two-armed randomised controlled trial will evaluate the effectiveness of a GP endorsed reminder in improving patient participation in the NHS Bowel Cancer Screening Programme (NHSBCSP). Up to 30 general practices in the West Midlands with a screening uptake rate of less than 50% will be recruited and patients identified from the patient lists of these practices. Eligible patients will be those aged 60 to 74, who have previously been invited to participate in bowel screening but who have been recorded by the Midlands and North West Bowel Cancer Screening Hub as non-responders. Approximately 4,380 people will be randomised in equal numbers to either the intervention (GP letter and duplicate FOBt kit) or control (no additional contact) arms of the trial.

The primary outcome measure will be the difference in the uptake rate of FOBt screening for bowel cancer between the intervention and control groups at 13 weeks after the GP endorsed reminder and duplicate FOBt kit are sent. Secondary outcome measures will be subgroup analyses of uptake according to gender, age and deprivation quartile, and the validation of methods for collecting GP, NHSBCSP and patient costs associated with the intervention. Qualitative work (30 to 40 semi-structured interviews) will be undertaken with individuals in the intervention arm who return a FOBt kit, to investigate the relative importance of the duplicate FOBt kit, reminder to participate, and GP endorsement of that reminder in contributing to individuals' decisions to participate in screening.

**Discussion:**

Implementing feasible, acceptable and cost-effective strategies to improve screening uptake amongst non-responders to invitations to participate is fundamentally important for the success of screening programmes. If this feasibility study demonstrates a significant increase in uptake of FOBt screening in individuals receiving the intervention, a definitive, appropriately powered future trial will be designed.

**Trial registration number:**

ISRCTN: ISRCTN86784060

## Background

Colorectal cancer (CRC) is the third most common cancer, and second leading cause of cancer death in the UK, with 35,000 diagnoses and 16,000 deaths per year [1]. It incurs an annual expenditure of more than £300 million in surgical, adjuvant and palliative treatment [2] which could be significantly reduced by earlier diagnosis through bowel cancer screening. The five-year survival rate for CRC is currently around 47% [3], lower than in other European countries [4,5]. Despite improvements in cancer survival over the past decade, the deprivation gap in survival for many cancers, including CRC, has been widening [6], and a key recommendation of the Department of Health's 2011 Cancer Reform Strategy is the development and implementation of strategies to facilitate earlier diagnosis of cancer, including the improvement and expansion of screening [7].

Biennial bowel screening using the Faecal Occult Blood test (FOBt) has been shown in randomised controlled trials (RCTs) to have the potential to reduce mortality from bowel cancer by 16% [8]. FOBt screening in the average risk asymptomatic population can detect bowel cancer at an earlier stage than would be the case through symptomatic presentation, meaning that treatment is more likely to be effective [9]. In addition to improved survival, the benefits of earlier diagnosis through screening include improved patient quality of life and reduced NHS treatment costs [10]. However, if these benefits are to be realised, high levels of screening uptake and continued adherence over time must be achieved and maintained in the eligible population.

In England, bowel cancer screening using FOBt was introduced in 2006 and has now been rolled out nationally, co-ordinated through the NHS Bowel Cancer Screening Programme (NHSBCSP). The programme aims to screen men and women aged between 60 and 74 every two years, using a guaiac faecal occult blood test. Evaluations carried out when the programme was in the pilot stage demonstrated relatively low rates of uptake (58.5% and 52% in the first and second rounds of screening respectively) [11,12].

Interventions to increase uptake are required, and in particular, the development of feasible and acceptable strategies to improve compliance with bowel cancer screening amongst non-responders. Several studies have investigated the role of various interventions in increasing compliance with cancer screening [13]. Patient interventions have typically focused on the effectiveness of invitations to screening in increasing uptake [14]; reminders sent to those who fail to respond to invitations for screening [15], and educational interventions to increase knowledge about a given screening programme [16,17]. Interventions aimed at providers have focused on reminders or prompts to general practitioners (GPs) to encourage individuals to undergo screening [18], and educational initiatives to maintain medical knowledge and levels of training in relation to bowel cancer screening [19].

Intervention studies conducted to date have typically failed to demonstrate significant improvements in compliance, or have had a greater impact on some sub-groups of the population than others [20], thus increasing inequalities in screening uptake further. Many have focused on opportunistic rather than population-based screening [21], and much research has been undertaken in the US, and as such may have limited applicability to the UK context. This indicates a need to consider alternative strategies to increase the uptake of bowel cancer screening in the asymptomatic population.

The involvement of GPs has been found to improve patient compliance with bowel cancer screening [19,21,22], and the uptake of FOBt is higher in groups that are sent a reminder to participate [14,15]. Doctors are typically cited as the most trusted profession in surveys of the public [23], and healthcare professionals (GPs in particular), are consistently rated as the primary source of information that patients seek regarding health and health services [24]. This existing research points to the importance of health professionals in supporting patients in making decisions about their healthcare, yet this research has largely been conducted outside of the UK, and no studies of interventions to increase the uptake of bowel cancer screening have been conducted since the introduction of the NHSBCSP in 2006. Furthermore, non-responders to previous invitations to participate in bowel screening have not been targeted as a specific group in whom rates of screening uptake may be increased. Despite GPs having minimal direct involvement with bowel cancer screening in the programme as currently designed, a potential strategy to improve participation in the NHSBCSP is a reminder from the GP, sent to non-responders to an initial invitation to participate in bowel screening. This approach has been found to be successful in increasing attendance for breast screening [25], yet the transferability of these findings to screening for bowel cancer is unknown, as it is aimed at a different target population. Furthermore, the factors contributing to an individual's decision to participate in bowel screening are likely to differ from those affecting decisions to participate in other cancer screening programmes.

### Study aim

The main aim of this feasibility study is to evaluate the effectiveness of a primary care based intervention (GP endorsed reminder and duplicate FOBt kit) on increasing the uptake of bowel cancer screening amongst non-responders to a previous invitation to participate.

## Methods/design

This feasibility study comprises a two-armed randomised controlled trial (n = 2190 in each arm) to evaluate the effectiveness of GP endorsement and a duplicate FOBt kit (compared with no additional contact) on improving the patient uptake of FOBt screening, and an embedded qualitative study in which semi-structured interviews will be undertaken with 30 to 40 individuals in the intervention arm of the trial who return their FOBt kit following the intervention.

### The NHS Bowel Cancer Screening Programme

Bowel cancer screening in England is organised through five regional screening Hubs which invite individuals to participate in screening - each regional Hub working with up to 20 local screening centres. The Hub sends an invitation and a leaflet about bowel cancer and the screening programme to all eligible patients registered with a GP in their region, followed by a FOBt kit around eight days later. Individuals are directed to complete the FOBt at home and return it to the screening centre for analysis. Those whose FOBt shows a positive result are then invited for further investigation through colonoscopy. If the Hub does not receive a completed kit within four weeks of sending it, a reminder is sent. After 13 weeks, the 'screening episode' is closed, and those who have not returned their kit are recorded as non-responders on the Hub database. In the Midlands, bowel cancer screening is co-ordinated by the Midlands and North West Bowel Cancer Screening Hub, based in Rugby.

### Study population

The study population will be adults aged between 60 and 74, on the patient list of a general practice where the bowel screening uptake rate is < 50%, who were invited to participate in the most recent round of bowel screening but who have not returned their FOBt within the 13 week 'screening episode' recorded by the Midlands and North West Bowel Cancer Screening Hub.

### Participant selection

Participant selection will be undertaken in two stages. First, the Midlands and North West Bowel Cancer Screening Hub will identify general practices in the West Midlands where the uptake of bowel cancer screening is lower than 50%. The contact details of these practices will be obtained via the MidReC (Midlands Research Practices Consortium) database, and all eligible practices will be contacted by the research team at the University of Birmingham and invited to participate, with general practice recruitment continuing until a total of up to 30 practices have been recruited. Once practices have been recruited, the Hub will identify all eligible screening non-responders aged 60 to 74 at each participating practice. Non-responders will be defined as those who have not responded to either the initial invitation to screening, or the 4-week reminder, within a period of 13 weeks after being invited.

### Inclusion criteria

Individuals aged between 60 and 74 years old, on the patient list at a general practice with a bowel cancer screening uptake lower than 50%, who have been recorded by the screening Hub as a screening non-responder no less than one month and no more than seven months previously.

### Exclusion criteria

Patients will be excluded from the trial if:

1. They have undergone an investigation (e.g. colonoscopy) within the past two years and/or are currently under surveillance following a previous colorectal abnormality

2. They have moved outside of the 60 to 74 year age range for screening in the time since their initial 13 week screening episode

3. They have contacted the Hub requesting not to be sent any information about bowel cancer screening, or FOBt kits

### Randomisation

For each participating general practice, the Hub will separate the names and details of the screening non-responders, determine any exclusions, and send anonymised details for eligible trial participants (each individual assigned a unique identifier) to the UK Clinical Research Network (UKCRN) accredited Primary Care Clinical Research and Trials Unit (PC-CRTU) at the University of Birmingham for patients to be randomised. Randomisation will be carried out using a computer generated randomisation algorithm, with patients randomised in equal numbers to the intervention or control arms of the study (n = 4,380). Block randomisation at the general practice level will ensure a balance of the two arms within each participating practice.

### Intervention Group

Patients in the intervention arm of the trial will receive a personalised letter recommending bowel cancer screening signed by their GP and a duplicate Hema-screen FOBt kit. The letter and duplicate kit will be sent to patients on behalf of the GP by the screening Hub. The same letter template will be used for all participating GP practices, but each GP will be able to modify the wording of the letter if there are particular issues that they would like to highlight to their patients. Patients recruited to the trial will be logged by the Hub database via a reopening of the individual's screening record, which would have been closed initially after the 13 week screening window that defined that individual as a non-responder.

### Control Group

Patients randomised to the control arm of the trial will receive no additional contact (current standard practice).

### Outcome measures

The primary outcome measure will be the difference in the uptake rate of FOBt screening for bowel cancer (i.e. the proportion of individuals who return a FOBt kit to the screening Hub) between the intervention and control group after 13 weeks.

Secondary outcome measures will include sub-group analysis of screening uptake rates following the intervention, according to age, gender and deprivation quartile, and the development and validation of methods and proformas for collecting information on GP, NHSBCSP and patient costs associated with the intervention. Such costs are anticipated to include the cost to the Hub of sending GP reminders to screening non-responders; costs associated with staff workload in sending reminders; time costs for participants, and costs to primary care if the intervention results in an increased number of patients contacting their GP to discuss bowel cancer screening.

### Outcome collection

13 weeks after initial contact (i.e. when an individual's screening episode is due to close), the Hub will log the number of FOBt kits returned by individuals in the intervention and control arms of the trial, and return anonymised lists of patients, denoted by a unique identification number, to the University of Birmingham for analysis. The member of hub staff collecting the outcomes data will be blinded to randomisation group to avoid bias.

Participating general practices will be asked to record prospectively all patient consultations about bowel cancer screening during the trial. In order to determine the relative effectiveness of retrieving information on consultations where screening was discussed from routine GP records (i.e. compared to prospective recording by the GP), a retrospective analysis of routinely collected GP records will be carried out in two of the participating practices. When the trial has ended, the screening Hub will produce a list of all patients who had been randomised within these two practices. Practice staff will be asked to inform the research team of the number of consultations relating to bowel cancer screening that have been carried out for these patients.

### Sample size

**Figure 1:**
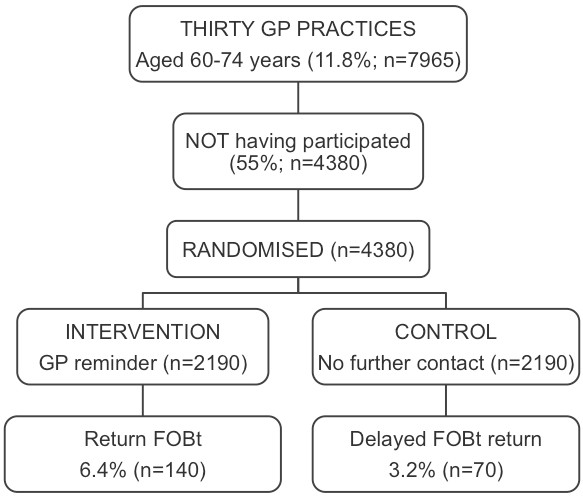
**Study schematic**.

General practices with a screening uptake rate of less than 50% will be purposively recruited. Assuming an average GP list size of 2,750 (information derived from the MidReC general practice database), with 11.8% of the population aged between 60 and 74 years old [26], and a bowel cancer screening participation rate of 45%, there will be 146 eligible individuals per practice (Figure 1). Thirty practices will provide 4,380 people for randomisation to the intervention or control arms of the trial. Assuming a screening uptake rate of 3.2% in the control arm (Midlands and North West Screening Hub recorded rate of delayed FOBt return amongst previous non-responders), a comparison of proportions test shows that a sample size of 934 in both the intervention and control groups is required in order to demonstrate a doubling of the uptake rate (from 3.2% to 6.4% or more) with 90% power at the 5% significance level. The larger number of patients (n = 2,190 in each group) has been chosen in order to enable sub-group analyses to be undertaken, and to account for any effects of nesting of patients within general practices should interim analyses demonstrate that such nesting is occurring.

### Qualitative study

Previous research exploring the patient factors associated with bowel cancer screening participation has provided limited information about significant sociodemographic and psychosocial factors, but the relative importance of these is unknown, and research has often yielded conflicting information [27]. Multi-factorial approaches, combining behavioural and educational strategies with healthcare interventions such as GP endorsed screening reminders are likely to be an important means of addressing the low uptake of CRC screening in the long term. Qualitative methodologies are a highly appropriate means of eliciting information about beliefs, attitudes and knowledge, and the ways that individuals justify their decision-making, particularly where the factors of concern may be unclear. Undertaking an embedded qualitative study will allow us to begin to understand the relative influence on individual decision-making of the three components of this intervention - receiving a reminder, receiving a duplicate FOBt kit, and endorsement of screening by an individual's GP.

#### Population

Approximately 30 to 40 individuals in the intervention arm of the study, who return their FOBt kit after receiving a reminder from their GP, will be recruited to participate in a semi-structured interview to investigate their reasons for returning the kit.

#### Recruitment

The Hub will send a letter inviting participation in a semi-structured interview to all individuals who have returned a FOBt kit following the intervention. A reply slip, on which patients can indicate their willingness to be interviewed, will be enclosed with the invitation letter, which will be returned to the University of Birmingham so that an interview can be arranged. All non-responders to an invitation to participate in an interview will receive one reminder from the screening Hub. Recruitment to the qualitative study will continue until data saturation has been reached, or until 30 to 40 interviews have been undertaken.

#### Feasibility

If the GP endorsed reminder is successful, we expect around 140 FOBt kits to be returned by patients randomised to the intervention arm of the trial. We aim to invite all patients in the trial intervention arm who returned a FOBt kit for interview, and conservatively estimate a response rate of 25% based on recruitment levels achieved in previous qualitative studies associated with bowel cancer screening uptake (Clements *et al. *personal communication). This would equate to approximately 35 individuals willing to participate in the qualitative phase.

#### Data collection

Interviews will take place either in a location of the participant's choice, or by telephone, and will be conducted by an experienced qualitative researcher. The topic guide will focus on attitudes and beliefs about bowel cancer screening, reasons for previous participation and/or non-participation in screening, and reasons for participation following the trial intervention. All interviews will be digitally audio-recorded and transcribed, and each will last approximately 60 minutes.

### Analysis

Trial data will be analysed on an intention to treat basis. The primary outcome measure (screening uptake in the intervention and control arms of the trial) will be analysed using a non-linear mixed logit model for dichotomous outcomes, accounting for practices as random effects. Secondary analyses will include comparison of screening uptake rates by sub-group on the basis of patient characteristics (age, gender, deprivation); whether or not the GP letter template was modified by a participating practice; previous bowel cancer screening behaviour, and according to the time period between an individual's first screening episode and the intervention. A multivariable prognostic model will be developed, incorporating interaction terms between factors. Decision tree analysis will also be undertaken, and the findings from these complementary analytical approaches will be cross-validated in order to identify factors predictive of delayed participation in the NHSBCSP [28]. Evaluating the potential influence of previous bowel screening behaviour on screening uptake rates within the feasibility work will inform the design of any future definitive trial with regard to establishing the need to stratify trial participants according to past screening behaviour prior to randomisation.

The characteristics of participating and non-participating general practices (i.e. those who were invited but who declined to participate) will be compared, using information derived from the MidReC general practice database in order to ascertain practice compliance rates and to investigate potential selection bias and confounders at the practice level. Comparisons will be made with regard to practice list size and other GP/practice-related factors (e.g. whether a practice is single-handed or has multiple partners; the socioeconomic deprivation profile of the practice catchment area, and the proportion of ethnic minority patients within the geographical area covered by the practice). These analyses will inform the design and methodology of any future definitive trial of the intervention.

Analysis within the qualitative study will be carried out by reading the interview transcripts and identifying emerging themes and categories. Each transcript will be independently analysed by two experienced qualitative researchers using thematic analysis. Themes arising from each interview will be compared in order to detect similarities and differences, and a constant comparative approach will be used, so that important themes arising from earlier interviews can be incorporated into a flexible topic guide for exploration in subsequent interviews.

### Data management

All activities undertaken by the Midlands and North West Bowel Cancer Screening Hub are covered by National Information Governance Board (NIGB, formerly the Patient Information Advisory Group, PIAG) approvals with regard to the handling of patient-identifiable data (Ref: PIAG 1-08(a)/2003).

No patient identifiable data will be seen by researchers outside of the screening Hub prior to consent for participation in the qualitative phase of the study. Any patient information (name, address) supplied by patients when returning a reply slip to the University of Birmingham indicating their willingness to participate in a semi-structured interview will be managed by the research staff with established procedures to ensure the confidentiality of those data and in accordance with applicable national and/or local regulations on personal data protection.

### Data monitoring

Although adverse events are not anticipated as part of this feasibility trial, it is possible that the intervention may increase the number of patients contacting the screening Hub in order to opt out of participation in any future rounds of bowel cancer screening. The Hub will inform the research team, at the end of the trial, of those individuals (anonymised, unique identifier only) who had contacted them to decline bowel cancer screening subsequent to receipt of the intervention.

An independent Data Monitoring Committee (DMC) will be formed to oversee the conduct of the trial, and will meet at regular intervals during the study. The DMC will comprise a statistician, a clinician, and a Specialist Screening Practitioner (SSP), and will evaluate interim outcomes and analyses to determine whether the study should be stopped for any reason.

### Ethical considerations

Ethical approvals have been obtained from South Birmingham Research Ethics Committee (Ref: 11/WM/0086), and R&D approvals have been obtained from University Hospitals Coventry and Warwickshire NHS Trust, Coventry Teaching Primary Care Trust and Birmingham and the Black Country RM&G Consortium Trusts (CSP Ref: 63560).

#### Consent to participate

Patients will be randomised without seeking consent. This approach is being taken to avoid the likely bias in screening uptake subsequent to the intervention that would result if participants were made aware of their involvement in the research study. Seeking consent from individuals identified as non-responders by the Hub would itself be an intervention; it would be impossible to ascertain whether any difference in uptake between the control and intervention arms was due to screening participation being prompted by an approach seeking consent for inclusion within the study, or because of the effect of the GP endorsed reminder and duplicate FOBt kit.

Written informed consent will be obtained from all participants in the qualitative phase of the study prior to interview.

## Discussion

There is a lack of evidence to support the implementation of effective interventions to reduce the burden of CRC by reducing delays in diagnosis or improving participation in screening. It is estimated that there would be 20,000 fewer deaths over the next 20 years if bowel cancer screening had an uptake of 60% [29]. The success and cost-effectiveness of the NHSBCSP depends on the achievement and maintenance of high screening uptake rates in the target population, and the development of feasible, acceptable and cost-effective strategies to improve compliance with bowel cancer screening offers the opportunity to improve programme delivery and to target population sub-groups who currently underuse the service.

If the intervention proves effective in the feasibility work, we envisage undertaking a definitive trial, which will be designed and powered based on the findings of the feasibility study. It is envisaged that the definitive trial may compare Hub and GP endorsement of bowel screening, with and without the inclusion of a duplicate FOBt, to determine the relative importance of different components of the intervention. It would also establish the cost-effectiveness of the intervention for different sub-groups, and assess the suitability of the intervention being rolled out nationally within the NHSBCSP.

## Trial Status

At the time of manuscript submission, recruitment to this trial had not yet begun.

## Competing interests

The authors declare that they have no competing interests.

## Authors' contributions

SW, SD and AC conceived the study and obtained research funding. SW, SD, SS, AC, RH and HD designed the trial and the intervention. SS will oversee delivery of the intervention, FDRH will contribute to the primary care aspects of the study. SC and LP will manage the trial and collect the data. LN, RH and SD will undertake data analyses. AC will undertake the qualitative work. SD wrote the first draft of this paper, which has been critically revised by all authors. All authors have seen and approved the final version of the manuscript.
